# Functional Outcome and Balance Compensation in Patients with Unilateral Vestibular Schwannoma After Surgical Treatment—Short- and Medium-Term Observation

**DOI:** 10.3390/jcm14020585

**Published:** 2025-01-17

**Authors:** Patrycja Torchalla, Agnieszka Jasińska-Nowacka, Magdalena Lachowska, Kazimierz Niemczyk

**Affiliations:** Department of Otorhinolaryngology Head and Neck Surgery, Medical University of Warsaw, Banacha 1a Str., 02-097 Warsaw, Poland; patrycja.torchalla@wum.edu.pl (P.T.); mlachowska.wum@gmail.com (M.L.); kniemczyk@wum.edu.pl (K.N.)

**Keywords:** vestibular schwannoma, cerebellopontine angle tumor, vestibular compensation, dizziness handicap inventory, dynamic computerized posturography, sensory organization test, vHIT

## Abstract

**Objective**: The aim was to evaluate vestibular function in patients with unilateral vestibular schwannoma before and in the short and medium term after surgical treatment to analyze vestibular compensation. The identification of the prognostic factors determining incomplete and slower balance recovery was assessed. **Methods:** Forty-five patients with unilateral vestibular schwannoma treated surgically through the middle cranial fossa and translabyrinthine approach were enrolled in this study. The data were collected in the period between April 2022 and August 2023. The clinical data, vestibular tests (video head impulse test, sensory organization test) and the dizziness handicap inventory (DHI) before and after surgery were evaluated. **Results:** One month after surgery, a temporary deterioration in the DHI results occurred (DHI total score before surgery 24.36 vs. one month after surgery 31.64); however, a significant increase was found only by analyzing the functional subscale (*p* = 0.0395) for the DHI functional, emotional and physical subscale results; in addition, the total score before and three months after the surgery did not differ significantly. No statistically significant differences between the preoperative sensory organization test and the test one month after the surgery were found, while a significant improvement in the vestibular parameters was observed three months after the surgery compared to the preoperative results (C5 0.0306, C6 0.0002, VEST 0.0294, COMP 0.0023). A negative correlation was found between the DHI total score and C5 (−0.3198, −0.3266), C6 (−0.3448, −0.46379), VEST (−0.3100, −0.3252) and COMP (−0.4018, −0.4854) one and three months after the surgery, respectively. A significant deterioration was found between the LSC gain results on the tumor side (*p* < 0.001) and on the healthy side before the surgery vs. one month afterwards (*p* = 0.0079) and before the surgery vs. three months afterwards (*p* = 0.0419). The middle cranial fossa or translabyrinthine approach had no influence on the postoperative results. **Conclusions:** In the postoperative period, vestibular compensation occurs spontaneously. The results show that the functional level deteriorates one month after surgery but then improves significantly three months after the surgery, which confirms that compensation occurs gradually. The DHI functional subscale results before surgery and three months afterwards did not differ significantly, which demonstrates that functional recovery after vestibular denervation should take place within that time. In the present study, no predictive factors for unsatisfactory functional postoperative outcomes were found.

## 1. Introduction

Vestibular schwannoma (VS) is a benign, slow-growing tumor originating from Schwann cells surrounding the vestibular nerve and located in the internal auditory canal and cerebellopontine angle [1]. The most common symptoms are sudden or progressive asymmetrical hearing loss and tinnitus, considered as red flags indicating further diagnostic investigation [2,3,4,5,6]. Non-vestibular schwannomas are relatively rare, with trigeminal and jugular foramen schwannomas being the most common with symptoms that are different from VS preoperative symptoms, such as sensory alterations in the trigeminal area, proptosis, neuropathic pain and diplopia [7,8,9].

Due to the slow growth, VS causes a gradual and progressive decline in vestibular function. However, it simultaneously results in gradual compensation by central adaptive mechanisms rather than an acute episode of spinning vertigo. Therefore, vestibular manifestation is less characteristic of VS but is presumed to be underreported. Nevertheless, vertigo is described by suffering patients as the most distressing symptom impacting their quality of life [6,7,8]. Vestibular symptoms are more often present in female patients, older patients and patients with larger tumors [10,11].

The treatment method choice for VS depends on factors such as tumor size, growth rate, severity of symptoms, patient preferences and comorbidities. The surgical removal of VS leads to complete unilateral vestibular denervation, resulting in vertigo, dizziness and balance instability immediately after surgery. This is due to the decompensation of the previously compensated situation affecting patients’ quality of life [12]. Some prior research confirms that patients who undergo active treatment such as surgery or radiotherapy suffer more often from vestibular symptoms compared with patients who remain under active surveillance [13], while other studies suggest that there is no evidence that treatment modality has better dizziness-related outcomes [14,15,16,17].

The recovery of balance is a slow process, developing progressively after surgical VS removal [18,19]. Vestibular compensation after tumor removal is a very interesting process, which may be affected by many factors. However, age, size, sex and vestibular rehabilitation seem to have no effect on the degree of long-term postintervention dizziness and balance recovery [14,20,21,22,23,24,25]. Despite numerous studies, the early phase of balance recovery remains poorly understood.

Detailed characteristics of the subjective functional outcome and objective vestibular tests results in patients with VS treated surgically would be helpful in better understanding balance compensation. Moreover, it could be essential in personalizing postoperative management.

Our study aimed to evaluate vestibular function before, a week after and one and three months after the surgical removal of unilateral vestibular schwannoma. We were interested in closely examining the early adaptation and vestibular compensation in patients with balance problems undergoing VS microsurgery. The secondary aim was to evaluate the functional disabling effects of vertigo or dizziness on everyday life based on the dizziness handicap inventory (DHI) and to compare them with vestibular test results. Moreover, we attempted to identify prognostic factors determining incomplete and slower balance recovery.

## 2. Materials and Methods

### 2.1. Ethical Considerations

The study was approved by the local Institutional Ethics Committee Review Board (AKBE/203/2022). Since this is a retrospective study, no informed consent was obtained.

### 2.2. Patients and Study Protocol Description

This retrospective study enrolled 45 patients diagnosed with unilateral VS confirmed with gadolinium-enhanced magnetic resonance imaging (MRI) and treated surgically. The data were collected from the period between April 2022 and August 2023. None of the patients received pre- or postoperatively specialized vestibular rehabilitation—the aim was to analyze in detail the effects of the automatic progressive implementation of central adaptive mechanisms resulting from postsurgical vestibular denervation with subsequent acute vestibular symptoms.

Before the surgery, a detailed medical history was taken from all the patients. All underwent routine ENT and otoneurological physical examinations and extensive audio-vestibular testing. Clinical symptoms were evaluated during the preoperative diagnostics. Data such as gender, weight, height, BMI, age at surgical treatment and the tumor size and side were collected. The tumor size was determined by measurement of the maximum tumor diameter on MRI T1-weighted scans with gadolinium enhancement.

An experienced otolaryngologist examined all the patients before the surgical treatment, on the first day of hospitalization, that is, 3–5 days before the surgery (BS); the values were then controlled at seven days (7D), one month (1M) and three months (3M) after VS removal. Before surgery (BS), the patients underwent audiological and vestibular tests, including pure-tone, speech and impedance audiometry, auditory brainstem response (ABR), acoustic otoemission, computerized dynamic posturography (CDP) with the sensory organization test (SOT) and the video head impulse test (vHIT). On the same day, the patients completed a Polish-validated version of the dizziness handicap inventory (DHI) questionnaire [26]. Seven days after surgery (7D), standard pure-tone audiometry and speech audiometry (only in patients after VS removal through the middle cranial fossa approach) and CDP with the SOT were performed. All the patients came for the follow-up visits one month (1M) and three months (3M) after surgery. During the follow-up visits, standard pure-tone and speech audiometry (only in patients after VS removal through the middle cranial fossa approach), CDP with the SOT, and the vHIT were performed. Each patient fulfilled the DHI during every follow-up appointment.

The present study focused on vestibular compensation after surgical treatment of unilateral VS. Consequently, we do not interpret the audiological results and postoperative facial nerve function in this study. The inclusion criteria for the current study were as follows: age above 18 years old, unilateral vestibular schwannoma treated originally by microsurgery and confirmed by the histopathological examination, no other previous known vestibular disorders and no postoperative complications affecting the management of postoperative balance recovery. Patients with bilateral VS tumors or neurofibromatosis type 2, preoperative radiosurgery, tumor recurrence after previous treatment, past medical history of any neurological problems, middle ear disorders and previous ear surgery, and patients with a diagnosis other than a VS histopathological diagnosis were excluded from this study. The present research focused on spontaneous vestibular compensation; therefore, patients who received pre- or postoperative rehabilitation were deliberately not included in the current study.

### 2.3. Surgical Treatment

Depending on the preoperative hearing levels and tumor size, the patients were assigned to the middle cranial fossa or translabyrinthine surgical approach. The same experienced otosurgeon operated on all the patients (KN).

#### 2.3.1. Middle Cranial Fossa Approach

A vertical incision is made in front of the auricle on the side where the tumor is present. The fascia from the temporal muscle is taken. Stabilizing sutures are placed on the skin and the temporal muscle is cut vertically. The surface of the temporal bone is exposed. A 3 × 3 cm temporal craniotomy is performed. The opening widens towards the base of the skull. The dura mater of the middle cranial fossa is dissected, stabilizing the position of the meninges and bleeding with oxycell if needed. A Fisch dilator is fitted. The superior petrosus nerve and the superior semicircular canal are located using the “blue line” identification method. The bottom of the internal auditory canal (IAC) is located and exposed along the entire course around the circumference of about 180°. The maters of the posterior cranial fossa above the IAC and below the superior petrosus sinus are exposed. The meninges of the posterior cranial fossa are opened medially from the internal auditory foramen, below the superior petrosus sinus. The meninges are opened along the IAC. The vestibular nerves in the fundus of the IAC are cut off. The tumor is dissected from the facial nerve and the cochlear nerve. The tumor is removed. On the upper wall of the IAC, layers of fascia from the temporal muscle and pieces of the temporal muscle are placed, sealing the base of the skull. Haemostasis is performed. Sutures are placed in the meninges. Restoration of the bone window is carried out.

#### 2.3.2. Translabyrinthine Approach

Fat tissue is collected from the abdominal wall. The wound is sutured in layers. A cut behind the ear of the affected side is performed. A wide antromastoidectomy is made with exposure of the meninges of the middle and posterior cranial fossa. The facial nerve is located. The structures of the posterior labyrinth are removed and the bone around the IAC is exposed (about 270°). The meninges of the posterior cranial fossa are incised. The tumor is exposed and dissected from the facial nerve. The tumor resection is performed. The opening in the meninges of the posterior cranial fossa is sealed with adipose tissue.

### 2.4. Analyzed Parameters

The study protocol with the analyzed parameters was described in detail in a previous article [27].

#### 2.4.1. CDP with SOT

The postural stability of each patient was evaluated with computerized dynamic posturography (CDP) with the sensory organization test (SOT). To assess the balance system, all six conditions (C1–C6) of the SOT, during which the patient’s task was to maintain an upright stance as stably as possible, were evaluated. Each condition (C1–C6) was examined three times (three trials), and each trial lasted 20 s. Each condition consisted of C1—eyes open, visual surround stable, platform stable, C2—eyes closed, visual surround stable, platform stable, C3—eyes open, visual surround moves, platform stable, C4—eyes open, visual surround stable, platform moves, C5—eyes closed, visual surround stable, platform moves and C6—eyes open, visual surround moves, platform moves. Each condition’s equilibrium score (ES) was calculated as the mean score of three consecutive trials. The equilibrium score is a percentage value representing the comparison of the patient’s body tilts with the appropriate limits of stability, determined on the basis of the average values obtained from healthy people of similar height, age and body weight to the patient. Parameters such as the somatosensory ratio (C2/C1), the visual ratio (C4/C1), the vestibular ratio (C5/C1), the visual preference ratio ((C3 + C6)/(C2 + C5)) and the composite score (the weighted average of the scores of all conditions) were analyzed [28,29]. The change in postural stability was followed with an SOT carried out before surgery and one week, one month and three months after surgery.

#### 2.4.2. vHIT

The video head impulse test (vHIT) examination included the standard protocol evaluating all six semicircular canals in three planes: the horizontal plane for the lateral canals, the plane oriented along the right-anterior–left-posterior (RALP) canals and the left-anterior–right-posterior (LARP) canals. In addition, a suppression head impulse paradigm (SHIMP) assessed the vestibulo-ocular reflex (VOR) inhibition. The VOR was assessed using the ratio of eye velocity to head velocity (gain) [30,31].

The present study focused on the VOR changes measured as a gain for the lateral semicircular canal on the affected side. A gain lower than 0.8 and higher than 1.2 is considered as abnormal [30,31]. The data from the vHIT were collected before surgery and one month and three months after surgery.

#### 2.4.3. DHI

Assessment of quality of life was evaluated with a self-report measuring using a Polish-validated version of the dizziness handicap inventory (DHI) [26]. The inventory consisted of 25 questions, which referred to the patient’s condition during the last month. The questions were divided into three subdomains to incorporate functional, physical and emotional impacts on disability. The patients were asked to complete the questionnaire preoperatively and one and three months postoperatively. According to the predefined responses, there were potential scores for each question as follows: 0 points for an answer “no”, 2 points for “sometimes” and 4 points for “always”. The maximum score was 100 (the worst possible) and the minimum was 0 points (the best possible). The DHI was interpreted according to the Whitney method [32]. Scores less than 30 were defined as a “light handicap”, between 31 and 60 as “average” and between 61 and 100 as “severe”. The patient’s equilibrium was considered as clinically significant improvement when the DHI outcome decreased by more than 18 points and as worsening when the DHI outcome increased by more than 18 points; a change in the DHI total score of below 18 points was considered clinically insignificant.

### 2.5. Statistical Analysis

Statistical analysis was conducted in the STATISTICA program (TIBCO Software Inc.: Palo Alto, CA, USA, 2017, version 13.3). The data were tested for normality, parametric and non-parametric criteria. Detailed statistical analysis was performed with the following tests. To evaluate changes between the pre- and postoperative results, the *t*-test was used for parametric data (DHI score, SOT results and vHIT gain value) and the Wilcoxon signed-rank test was used for non-parametric data (saccades presence in the vHIT). Spearman’s rank correlation and Pearson Correlation were used to analyze the relationships between the clinical data. The level of statistical significance was set at *p* = 0.05. To identify the prognostic factors characteristic of incomplete vestibular compensation, the study population was divided into three groups depending on the DHI outcome as described below and differences in the clinical features between these subpopulations were analyzed.

## 3. Results

### 3.1. Patients’ Characteristics

Forty-five patients diagnosed with unilateral VS (23 on the right side, 22 on the left side) based on MRI scans and histopathological results were enrolled in this study. The mean age of the patients at the time of surgery was 52.09 ± 10.98 years old (min. 34.00, max. 77.00). There was a slight female predominance, with 24 females and 21 males. According to the tumor size by the Koos grade, there were 15 patients with a grade I tumor, 21 with grade II, 5 with grade III and 4 with grade IV [33,34].

The tumor size varied from 4.70 to 44.00 mm (mean 15.81 ± 8.56 mm). The mean duration of symptoms varied from 1.00 to 30.00 years (mean 4.04 ± 5.12 years). The most common and at the same time most frequently described as the first presenting symptom was progressive unilateral hearing loss in 20 patients (44.44%) and tinnitus in 14 (31.11%). As for the dizziness problems, four patients (8.89%) reported vertigo and two imbalance (4.44%). In two cases (4.44%), the first symptom was ear fullness, while a headache was reported in one patient (2.22%) and deterioration in speech understanding in two patients (4.44%).

All the patients were treated by microsurgery. Surgical approaches included the middle cranial fossa and translabyrinthine approach in 21 (46.67%) and 24 (53.33%) patients, respectively. 

### 3.2. DHI Results

**Figure 1:**
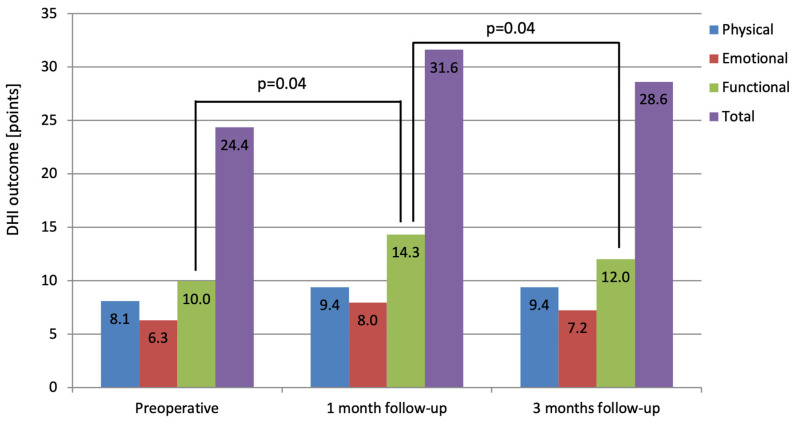
The results of the dizziness handicap inventory (DHI) in patients with vestibular schwannoma before and one and three months after surgical treatment. Paired *t*-test for parametric data was used to evaluate changes in the subsequent results. The *p*-value represents statistical significance.

The DHI scores for each period are illustrated in Figure 1 and summarized in Table 1. The detailed data are presented in Table S1. The mean DHI total score before the surgery was 24.36. The mean scores for the preoperative subscales were 8.09, 6.31 and 9.96 for the physical (P), emotional (E) and functional (F) subscales, respectively (Table 1, Figure 1).

At the follow-up one month after the surgery, the mean DHI total score was 31.64; at the next follow-up, three months afterwards, it was 28.62. After the VS removal, the physical subscale scores were 9.38 and 9.38, the emotional subscale scores were 7.96 and 7.24, and the functional subscale scores were 14.31 and 12.00, one month and three months afterwards, respectively. Statistically significant differences were found between the DHI functional subscale before vs. one month after (increase, *p* = 0.0395) and between one month vs. three months after (decline, *p* = 0.0352) the surgery. Other differences in the DHI scores between before and after the VS resection were insignificant (Figure 1).

Based on the DHI scores, a light handicap (DHI ≤ 30) was observed in 62.22%, 57.78% and 66.67% of the patients before surgery and one and three months after surgery, respectively. An average handicap (DHI 31–60) was described in 28.89%, 24.44% and 20% of the patients before surgery and one and three months after surgery, respectively. A severe handicap (DHI 61–100) was seen in 8.89%, 17.78% and 13.33% of the patients before surgery and one and three months after surgery, respectively (Table S2 Panel A).

A clinically significant balance deterioration, considered as an increase of a minimum of 18 points compared to the results obtained before surgery, was observed in 28.89% and in 31.11% of the patients one and three months after surgery, respectively. Moreover, a clinically significant improvement in balance measured by a decrease of 18 points and more, compared to the results before the surgical removal of the VS was obtained in 13.33% and 15.56% of the patients after one and three months of observation, respectively (Table S2 Panel B).

### 3.3. SOT Results

**Figure 2:**
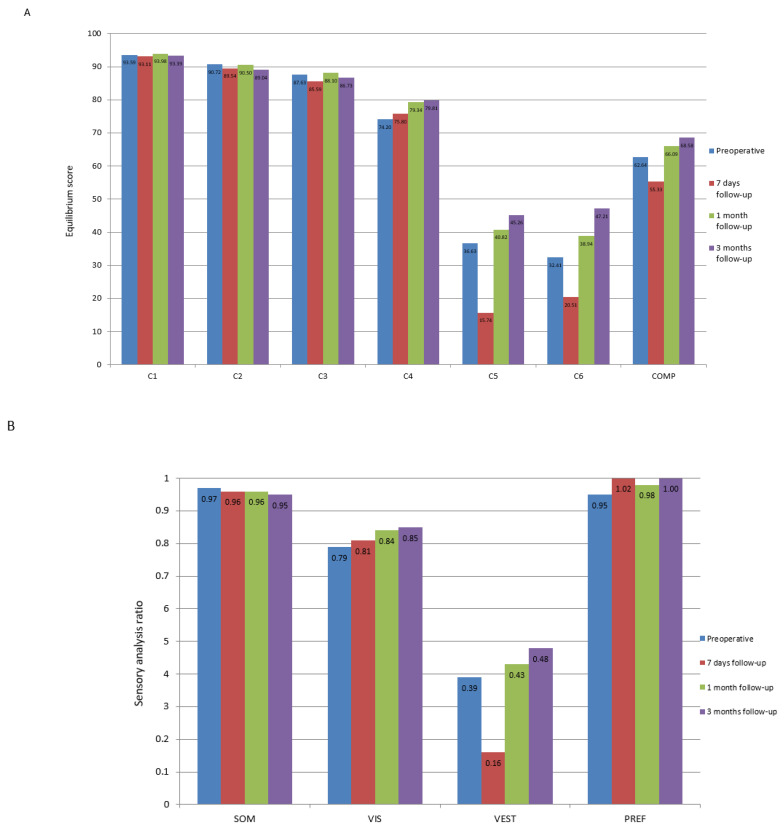
The results of the computerized dynamic posturography—sensory organization test (SOT) in patients with vestibular schwannoma before and after surgical treatment. (**A**) represents average results of each of the conditions tested in SOT and general equilibrium score. (**B**) represents results of the sensory analysis. The *p*-value representing statistical significance is presented in Table 2. C1–C6—condition 1 to condition 6, SOM—somatosensory, VIS—visual, VEST—vestibular, PREF-preference, COMP—composite.

The SOT results are shown in Table 1 and Table S1 and Figure 2. Statistically significant differences between the pre- and postoperative results are presented in Table 2.

### 3.4. vHIT Results

The gain in the LSC on the affected and contralateral side in the vHIT was evaluated and is presented in Table 1 and Table S1. The mean gain in the LSC on the tumor side at the baseline was 0.97. In total, 17.78% of the patients achieved a gain of less than 0.8 before the surgery. One month after the VS removal, the mean gain was 0.52. A total of 82.22% of the patients had a gain < 0.8 one month after the surgery. Three months after the surgery, the gain was 0.56. A gain < 0.8 was found in 77.78% of the patients three months after the surgery.

The mean gain in the LSC on the contralateral side before the surgery was 1.16. One month after the surgery, the gain was 1.05, while three months afterwards, it was 1.06. A gain < 0.8 on the healthy side was found in 4.44%, 6.67% and 11.11% of the patients before surgery and one month and three months afterwards, respectively.

**Figure 3:**
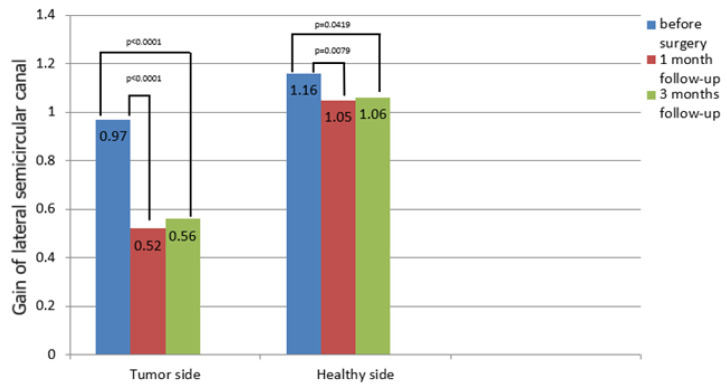
Gain in lateral semicircular canal of the tumor side and healthy side in video head impulse test before and 1 month and 3 months after surgery. The *p*-value represents statistical significance.

A significant deterioration was found between the LSC gain results on the tumor side before the surgery vs. one month afterwards (*p* < 0.001) and before the surgery vs. three months afterwards (*p* < 0.001). Moreover, a significant deterioration was also found between the gain results on the healthy side before the VS removal vs. one month afterwards (*p* = 0.0079) and before vs. three months afterwards (*p* = 0.0419) (Figure 3).

### 3.5. Correlations Between Clinical Data and DHI Scores

No significant correlations were found between patients’ age and the DHI results before (DHI total score *p* = 0.3624; P subscale *p* = 0.1244; E subscale *p* = 0.7183; F subscale *p* = 0.5724), one month after (DHI total score *p* = 0.8391; P subscale *p* = 0.8665; E subscale *p* = 0.6228; F subscale *p* = 0.8068) and three months after the surgical VS removal (DHI total score *p* = 0.9448; P subscale *p* = 0.8187; E subscale *p* = 0.634; F subscale *p* = 0.946).

There was no significant correlation between the maximal tumor diameter and the DHI results before (DHI total score *p* = 0.1972, P subscale *p* = 0.4017, E subscale *p* = 0.077, F subscale *p* = 0.1705) and one month after the surgery (DHI total score *p* = 0.0963, P subscale *p* = 0.1763, E subscale *p* = 0.1668, F subscale *p* = 0.0666). There was a significant negative correlation between the maximal tumor diameter with a total DHI score (*p* = 0.0383) and emotional (*p* = 0.0229) and functional (*p* = 0.0225) DHI subscales scores three months postoperatively. However, a correlation between the P subscale after 3 months was not found (*p* = 0.2433).

### 3.6. Correlations Between Clinical Data and Vestibular Test Results

Correlations between the patients’ clinical data and the posturography results were analyzed. A significant negative correlation was found between the patients’ age and the SOT results before the surgery for C5 (*p* = 0.0001), C6 (*p* = 0.0005), VEST (*p* = 0.0001) and COMP (*p* < 0.0001).

A significant negative correlation was found between age and the SOT results in C5 (*p* = 0.0188), C6 (*p* = 0.0415) and VEST (*p* = 0.0208) one month after the surgical treatment. No correlation was found in COMP (*p* = 0.0689) after one month.

No correlation was found between the maximum tumor diameter and the SOT results before surgery (C5 *p* = 0.9675; C6 *p* = 0.4681; VEST *p* = 0.9323; COMP *p* = 0.8538).

No significant correlation was found between the tumor size and the SOT results (C5 *p* = 0.9953; C6 *p* = 0.7408; VEST *p* = 0.9366; COMP *p* = 0.8942) one month after the surgical tumor removal.

Three months after the surgery, a significant negative correlation was found between the patients’ age and C5 (*p* = 0.0204), VEST (*p* = 0.0246) and COMP (*p* = 0.048). No correlation was found in C6 (*p* = 0.1849). The maximum tumor size had no influence on the C5 (*p* = 0.0524), C6 (*p* = 0.4067) and COMP (*p* = 0.0648) scores three months after the surgery. However, a positive correlation was found in the VEST results (*p* = 0.0457).

The relationships between the clinical data and vHIT results were analyzed. No significant correlations were found between age and the gain in the LSC on the tumor side before (*p* = 0.0536) and after the surgical removal (1 month *p* = 0.37; 3 months *p* = 0.1242).

There was a significant negative correlation between the maximum tumor diameter and the gain in the LSC on the affected side before the surgery (*p* = 0.024). No significant correlation was found between the tumor size and the gain in the LSC during the follow-up visits after the surgical VS removal (1 month *p* = 0.225; 3 months *p* = 0.184).

### 3.7. Correlations Between DHI Questionnaire and Vestibular Test Results

Relationships between the DHI questionnaire scores and posturography results were analyzed and are presented in Table 3. The results of the DHI scores and vHIT results were also compared (Table 4).

There was no significant correlation between the DHI results and LSC gain in the vHIT before the surgery and 1 month and 3 months after the surgery.

### 3.8. Correlations Between vHIT and Posturography Results

Before the surgery, the LSC gain in the vHIT correlated with the C5, VEST and COMP results of the SOT, while no correlation was found between the LSC gain and C6. One month after the surgery, the LSC gain correlated negatively with COMP only. Three months after the VS surgery, a significant negative correlation was found between the LSC gain and C5 and VEST parameter (Table 5).

### 3.9. Differences in Clinical Characteristics and Vestibular Test Results in Reference to the Postoperative Functional Level

The surgical approach (middle fossa or translabyrinthine approach) presented no influence on the postoperative results one and three months afterwards using the DHI (total, P, E, F subscales), vHIT (gain in LSC) and SOT (C5, C6, VEST, COMP).

In the present study, the patients were divided into three groups according to the changes in the DHI three months after the surgery compared to the presurgical results (Table S1 Panel B). The first group consisted of seven patients (15.56%) where the total DHI score decreased by least 18 points or more. The second group of 24 patients (53.33%) had no clinically significant change in the DHI total score (DHI without changes of 18 points) and the third group of 14 patients (31.11%) had increases of at least 18 points in their DHI total score. We were looking for any specific factor that could influence the early postoperative period and cause a better or worse DHI score during the three-month follow-up period. Any significant statistical difference was detected in gender, age, weight, height, surgical approach, maximum tumor diameter, Koos grade and mean duration of symptoms in these three different groups of patients. What is more, in the aforementioned groups, any of the presurgical or postsurgical gain results in the LSC in the vHIT and any parameter in the SOT (C5, C6, SOM, VIST, VEST, PREF, COMP) obtained during our follow-up (before surgery, 7 days after surgery, 1 month after surgery, 3 months after surgery) did not have an influence on DHI changes and did not differ significantly between these three groups of patients.

## 4. Discussion

### 4.1. Postoperative Functional Assessment Using DHI Questionnaire

Directly after vestibular schwannoma removal, patients may suffer from vestibular symptoms. The recovery after acute vestibular disorder may last weeks or months. However, the clinical course is individually variable and unpredictable. A gradual improvement in symptoms is seen due to vestibular compensation and progressive adaptation of the vestibular nuclei [35]. Despite this, instability and imbalance may stay persistent in some cases. Vestibular tests are very helpful in the presurgical evaluation of the vestibular system in patients with VS and of the postoperative follow-up.

In our study, one month after the surgery, there was a noticeable deterioration in the functional DHI subscale compared to that before the surgery, which is typical of acute vestibular denervation. Three months after the surgery, there was a significant improvement in the functional subscale compared to the results obtained one month after the surgical treatment, and the results were similar to the results before the surgery. Similarly, the DHI total score results increased one month after the surgery, but they were not significant. The results then improved (decreased) three months later, reaching the presurgical level, showing the patients’ dizziness handicap before and three months after the surgery did not differ significantly. This suggests that vestibular compensation generally occurs spontaneously with daily life activity during that time.

Humphriss et al. [22] collected data from the DHI retrospectively in 100 patients with VS who underwent translabyrinthine tumor removal. All of their patients had been prescribed generic vestibular rehabilitation exercises. In this group, tumors smaller than 1.5 cm, between 1.5 cm and 4.4 cm, and larger than 4.4 cm were found. Contrary to our study, they found a significant difference between the preoperative and three-month scores (increased DHI) and the preoperative and twelve-month scores (increased DHI). There was no significant difference between the 3-month and 12-month scores in this group of patients. This may confirm that vestibular compensation appears within first 3 months after vestibular deafferentation, so there is no significant change in the DHI scores when comparing the 3- and 12-month results. What is more, in the aforementioned study, when using the 18-point criterion, 73% of the patients had no significant changes in DHI scores 3 months postoperatively. For most of the patients, the DHI scores measured 3 months after surgery did not worsen. For those patients, if a worsening in the DHI scores appeared, it occurred in the first 3 postoperative months and not after that. In our group, taking into consideration the 18-point criterion, no significant changes in DHI were observed in 53.33% of the patients when comparing the results three months after the surgery and before the surgery. While the trend described above suggests functional compensation, it should also be noted that in more than half of the patients, the handicap level caused by the balance disorders did not change. The average DHI total results before and after the surgery are in the range of mild severity, which confirms that balance problems are not the most bothersome complaint reported by patients with VS. Moreover, no significant changes in DHI were observed in 77.78% of the patients when comparing the results one and three months after surgery. Both studies confirm the importance of follow-up evaluation of patients after VS surgery, as the early postoperative results may determine the final clinical outcome.

Godefroy et al. [20] analyzed DHI scores in patients with small intracanalicular VS with no extrameatal growth who underwent a translabyrinthine approach and vestibular rehabilitation. They analyzed the scores before and three and twelve months after the surgery. All the patients before the surgical treatment had persistent vertigo or disequilibrium classified as grade IV according to Kanzaki et al. [36]. Similarly to our study, the total DHI scores showed no significant difference before and three months after the surgery. Still, significant differences were found between the preoperative scores and results after 12 months. When the 18-point criterion was used, the DHI scores were significantly improved in 30% of the patients and no significant improvement was observed in 70% of the patients at 3 months post-surgery. There were no significantly worse DHI scores at 3 or 12 months after surgery. At 12 months postoperatively, 88% of the patients had a significant improvement in DHI scores when compared with the preoperative scores. According to that study, for most patients with severe preoperative balance disorders, a significant DHI improvement can be observable 3 months after the VS removal. This suggests that observation longer than 3 months needs to be taken into consideration, especially in patients with persistent vertigo and disequilibrium before surgery. Presumably, the long-term compensation pattern described in that study may result from the different characteristics of the group, with an average preoperative DHI result of 51.3 in comparison to 24.36 in our population. Moreover, a small intracanalicular VS with no extrameatal growth may be characterized by a unique manifestation of balance compensation. Nevertheless, further studies with longer follow-up are necessary to better investigate this subject.

Relations between DHI scores and age differ in the literature. There are studies in the literature that did not find a correlation between age and DHI scores in patients after surgical VS removal [21,22,25,37,38]. In Humphriss et al.’s article [22], age was not found to be a significant factor affecting the change in the DHI score, nor was this the case in the current study. Surprisingly, a patient’s age may not be a significant single predictive factor for subjective clinical outcome after the surgery. However, Carlson et al. [39] analyzed multivariable correlations between postoperative DHI scores and clinical data and described that before any treatment for VS (observation, microsurgery, stereotactic radiosurgery), increasing age, a larger tumor size (20–30 mm), the presence of dizziness before treatment, increasing frequency of headaches at the time of the survey and moderate or severe headaches at the time of the survey were statistically significantly associated with poorer DHI total scores in a multivariable setting. Our study found a significant negative correlation between the tumor size and the total, emotional and functional scores in the DHI results three months postoperatively. Interestingly, the tumor size affects the DHI total, emotional and functional scores three months after the surgery despite having no correlation with the preoperative vestibular symptoms. The results of the present study may indicate that the slow growth of larger VSs enables subsequent vestibular compensation during the natural disease course, following the same trend as the smaller tumors. Presumably, the duration of the tumor growth would be more important than its size; however, the duration of the presurgical symptoms did not influence the DHI results pre- and postoperatively in our study.

In the present study, we divided the patients into three subpopulations, depending on the postoperative DHI result change (increase of 18 points, decrease of 18 points, without clinically significant change). We defined the subpopulation with a postoperative increase in the DHI result of at least 18 points as patients with unsatisfactory functional compensation. To identify the predictive factors associated with poor functional compensation, we were looking for differences between the three aforementioned subpopulations. The aim was to identify a subpopulation that cannot achieve spontaneous functional compensation. These patients could be referred to a specialized vestibular therapy unit. However, no statistically significant differences were found between these groups; thus, no specific factor influencing the postoperative changes in the DHI total score was found among the patients’ clinical data, tumor size or preoperative vestibular tests results. Moreover, no predictive factors were found among the early postoperative results (one week or one month after the surgery). This needs further study and longer observation of patients to identify the group of patients at risk of slow compensation, who may need personalized postoperative management and advanced postsurgical rehabilitation. It would be crucial to extend the observation of the patients who achieved unfavorable results in the postoperative DHI scores to check whether the deterioration in the DHI scores is persistent or changes over a more prolonged time than the three months after surgical treatment. Further research is needed on long-lasting clinical outcomes.

### 4.2. The Role of Computerized Dynamic Posturography in Evaluation of Postoperative Vestibular Compensation

According to Parietti et al. [40], a satisfactory recovery of balance control after vestibular schwannoma surgical removal can be obtained within three months of surgery. They proved this using SOT assessments—the SOT was performed on 36 patients with unilateral vestibular schwannoma before surgical treatment by the translabyrinthine approach, three months, six months and twelve months after surgery. For the equilibrium scores and ratios, the values before the surgery were lower than those in the three other stages for the main parameters. The scores for C3, C4, C5, C6 and COMP obtained before the surgery were significantly lower than those obtained 3 months after surgery. For C4, C5, COMP and VEST, the values 6 months after the surgery and 12 months after the surgery were higher than those 3 months after the surgery. In our study, the SOT was repeated on every follow-up visit as the results could be reliably compared to the previous tests and did not cause patient discomfort in the early postoperative period. Compared to the preoperative examination, the results of the vestibular tests (C5 and C6) and VEST and COMP parameters were significantly worse on the seventh day after the surgery, which can be explained by acute vestibular loss. The fact that there were no statistically significant differences between the preoperative results and those one month after the surgery demonstrate vestibular compensation that continues within the next months, as illustrated by the significant improvement in the C5, C6 and VEST results three months after the surgery compared to the preoperative results. Analyzing the general balance level using the COMP parameter, the results presented a similar trend, but a significant improvement in relation to the pre-treatment examination occurred as early as one month after the surgery. The VIS result, assessing the ability to use visual information to maintain balance, did not deteriorate on the seventh day after the surgery, but a significant improvement in that parameter was observed one and three months after the surgery. Since the VIS parameter improved after the tumor removal, an important role of vision in the compensation after acute vestibular denervation may be suspected.

Several correlations found between the patient’s age and the pre- and postoperative SOT results confirms a deterioration in balance progressive with age, which is consistent with the literature [41]. Some authors agree that age is related to a decline in postural control and cognitive processes, but this does not impair the postural compensation mechanisms [42].

Moreover, subjective dizziness and vertigo in vestibular schwannoma patients can be associated with postural instability observed in posturography, as numerous associations between the DHI and SOT results were found in the present study. Before the surgery, statistically significant correlations were found between the general balance level evaluated by the COMP parameter and the DHI P subscale. Moreover, after the surgery, correlations between the vestibular tests (C5, C6 and VEST) and COMP parameter and the DHI results were found, as presented in the Table 3. The results mentioned above confirm that posturography can be used to objectify the vestibular symptoms reported by patients with VS and after the surgical treatment, and that this can be useful in evaluating the functional compensation. As the examination is non-invasive and well-tolerated by patients, it can be used as a follow-up method.

The SOT results are presented in a graphical format that can be shown to the patients and is quite easy to understand. Thus, improving the SOT results may positively impact the psychological aspect of the patients’ recovery and facilitate their return to daily activities, which is very important to the functional improvement in patients with vestibular disorders [43].

### 4.3. The Application of vHIT in Pre- and Postoperative Evaluation in Patients with VS

The vHIT is used to detect lesions in all the semicircular canals, and the test’s sensitivity to VS is 80%, which suggests that the vHIT could be used as a screening tool for VS, owing to its convenience [44]. It is an easy tool to examine vestibulopathy and the test should be performed in every case, as suggested in the literature [5,45]. The vHIT can evaluate the origin of VS through VOR, detect the severity of function loss in the affected semicircular canal and monitor the progression of VS [40]. Patients with VS may not present typical symptoms of vertigo but still have a significant decrease in the gain shown in the vHIT results [44]. In the present study, the preoperative LSC gain correlated with the tumor size, which confirms a progressive loss of vestibular function occurring with the growth of the VS. The tumor maximum diameter and grade in Koos classification were correlated to the gain in the preoperative vHIT, suggesting that the growing tumor mass affects the vestibular function despite the lack of correlations with the subjective functional scales. No significant correlations were found between age and the presurgical gain in the LSC on the tumor side, which is similar to other studies [46,47,48,49]. What is more, no significant correlations were found between the LSC gain and DHI results, which was also shown in previous studies [49]. Moreover, significant correlations were found between the LSC gain and SOT results (C5, VEST, COMP) before the surgery, indicating that the vHIT can be used to assess the balance system function as a screening tool in patients with VS. As mentioned, the vHIT device is light and portable; what is more, it is relatively cheap compared to other vestibular tests. As an easy tool, it can be used during follow-up visits to the outpatients clinic to evaluate the balance compensation, especially in departments without CDP access.

A significant deterioration in the LSC gain was present after the surgery, which confirms the loss of VOR after vestibular denervation. The postoperative vHIT results did not correlate with the tumor size, which is consistent with our expectations, as complete vestibular denervation occurred in patients with small and big tumors as well. Moreover, after the surgery, a significant deterioration was also found on the contralateral side—during head movement to the healthy side. Presumably, this may be due to the fact that both vestibules are involved in a normal VOR bilaterally. Thus, complete denervation of one vestibule results in subtle VOR disruption during the head movement toward the healthy side.

Surprisingly, in the present study, the tendency of correlations between the vHIT and SOT results changed after the surgery, as some negative correlations were found between the LSC gain and balance scores. One month after the surgery, a negative correlation was found between the LSC gain and COMP parameter and 3 months after surgery a negative correlation was also found between the LSC gain and C5 and VEST. Nevertheless, these correlations may be explained by the decrease in the vHIT gain caused by vestibule denervation, and it may not be associated with the decrease in the SOT results as the compensation occurs.

Regarding the vHIT test, a limitation of our study was that only the lateral semicircular canal (the lateral vestibulo-ocular reflex) and thus the superior vestibular nerve were considered. Theoretically, a tumor arising from the inferior vestibular nerve could give a false-negative outcome.

Rahne et al. [50] have shown that using vHIT amplitudes alone has a rather small discriminatory power between IVN and SVN tumors. Despite this, the vHIT overall shows a large sensitivity in diagnosing VS [31], but the prediction of tumor origin with the vHIT alone would not be precise enough. Although the majority of VSs originate from the inferior branch of the vestibular nerve, they exert pressure on the superior branch and occupy the internal auditory canal. Thus, the vestibular function can be disturbed in the area supplied by both branches. The protocol evaluating the LSC is the easiest part of the vHIT examination to perform, in comparison to the RALP and LARP test, which is often disturbed by artifacts. Nevertheless, extended studies in the future should examine the vestibulo-ocular reflex of all semicircular canals to better understand the relationships between the vHIT result and functional outcome in patients with VS.

According to Tranter-Entwistle et al. [49], the DHI score could not be predicted from the vHIT gain, which is similar to our results.

Consequently, in our opinion, both objective vestibular tests and subjective questionnaires are essential to evaluate balance system pre- and postoperatively.

## 5. Study Limitations

The current study, as retrospective research, has several limitations that should be acknowledged. First of all, the collected data were obtained only from patients treated surgically through the middle cranial fossa and translabyrinthine approach. The retrosigmoid approach was not included in the current study and nor were radiosurgery cases. The group consisted of only 45 patients and the observation period was established as the short and medium term up to 3 months. Further studies with a longer observation period, preferably up to one year, are necessary to obtain long-term results for balance compensation and for identifying and qualifying the appropriate group of patients for pre- or postoperative vestibular rehabilitation so they can achieve better balance recovery. Moreover, additional vestibular tests like the VEMPs and RALP/LARP vHIT protocol evaluating the inferior vestibular nerve could be important in a better understanding of vestibular compensation. In the presented study, no dedicated vestibular rehabilitation was implemented. Consequently, in the future, for a complete interpretation of the functional outcomes, a comparison with patients undergoing specialized rehabilitation will be crucial.

## 6. Conclusions

The findings of this detailed analysis could be important to a better understanding of vestibular compensation. Our retrospective study included patients who did not undergo dedicated pre- or postoperative vestibular rehabilitation. Thus, the results describe spontaneous compensation. The results show that the functional level deteriorates one month after complete vestibular denervation but then improves significantly three months after the surgery, which confirms that compensation occurs gradually. The DHI functional subscale results before the surgery and three months afterwards did not differ significantly, which demonstrates that functional recovery after vestibular denervation should take place within that time. In this way, our study proved that vestibular compensation is a spontaneous process in most patients’ everyday lives. This is an important conclusion considering the limited access to postoperative vestibular rehabilitation in many centers.

The results may indicate that the slow growth of larger VSs enables subsequent vestibular compensation, following the same trend as smaller tumors. In the present study, no predictive factors for unsatisfactory functional postoperative outcomes were found. However, the tumor size impacts on the postoperative results, so probably this group of patients may require dedicated rehabilitation to obtain better compensation. This might be helpful for clinicians to personalize the pre- and postoperative management of patients with VS. In addition, this study may be helpful for clinicians to manage VS patients before and after surgical removal.

## Figures and Tables

**Table 1:** Average pre- and postoperative results of the analyzed patients with unilateral vestibular schwannoma before and after surgical treatment.

		Before the Surgery	7-Day Follow-Up	1-Month Follow-Up	3-Month Follow-Up
Dizziness Handicap Inventory (DHI)	total score (0–100 points)	24.36 ± 24.35		31.64 ± 27.65	28.62 ± 27.22
	P subscale (0–28 points)	8.09 ± 7.75		9.38 ± 8.18	9.38 ± 7.83
	E subscale (0–36 points)	6.31 ± 7.65		7.96 ± 9.27	7.24 ± 9.39
	F subscale (0–36 points)	9.96 ± 10.26		14.31 ± 11.68	12.00 ± 11.26
Video Head Impulse Test (vHIT)	LSC gain tumor side	0.97 ± 0.29		0.52 ± 0.27	45.26 ± 0.30
	LSC gain healthy side	1.16 ± 0.26		1.05 ± 0.21	47.21 ± 0.22
Sensory Organization Test (SOT)	C5 (0–100)	36.63 ± 22.39	15.74 ± 19.81	40.82 ± 25.33	45.26 ± 23.46
	C6 (0–100)	32.41 ± 24.41	20.51 ± 21.61	38.94 ± 24.98	47.21 ± 23.62
	SOM ratio (C2/C1)	0.97 ± 0.03	0.96 ± 0.05	0.96 ± 0.03	0.95 ± 0.07
	VIS ratio (C4/C1)	0.79 ± 0.16	0.81 ± 0.10	0.84 ± 0.11	0.85 ± 0.14
	VEST ratio (C5/C1)	0.39 ± 0.24	0.16 ± 0.21	0.43 ± 0.27	0.48 ± 0.25
	PREF ratio [(C3 + C6)/(C2 + C5)]	0.95 ± 0.19	1.02 ± 0.21	0.98 ± 0.19	1.00 ± 0.22
	COMP score (0–100)	62.64 ± 11.75	55.33 ± 9.80	66.09 ± 11.66	68.58 ± 13.63

LSC—lateral semicircular canal; C—condition; SOM—somatosensory; VIS—visual; VEST—vestibular; PREF—visual preference; COMP—composite.

**Table 2:** The results of the computerized dynamic posturography—sensory organization test (SOT) in patients with vestibular schwannoma before and after surgical treatment.

SOT Parameter	Before vs. 7 Days After Surgery	Before vs. 1 Month After Surgery	Before vs. 3 Months After Surgery	7 Days vs. 1 Month After Surgery	7 Days vs. 3 Months After Surgery	1 Month vs. 3 Months After Surgery
C1	0.2476	0.4893	0.6805	0.0394 *	0.4544	0.1140
C2	0.0785	0.7456	0.0185 *	0.0770	0.6476	0.0995
C3	0.2192	0.6504	0.6495	0.0843	0.6465	0.4664
C4	0.4716	0.0330 *	0.0389 *	0.0051 *	0.0430 *	0.8000
C5	<0.0001 *	0.3417	0.0306 *	<0.0001 *	<0.0001 *	0.1753
C6	0.0091 *	0.0535	0.0002 *	<0.0001 *	<0.0001 *	0.0007 *
SOM	0.3687	0.2139	0.0687	0.7936	0.4775	0.3138
VIS	0.3412	0.0388 *	0.0339 *	0.0308 *	0.0574	0.6239
VEST	<0.0001 *	0.3553	0.0294 *	<0.0001 *	<0.0001 *	0.1492
PREF	0.1115	0.3996	0.2298	0.2550	0.7815	0.5407
COMP	0.0001 *	0.0446 *	0.0023 *	<0.0001 *	<0.0001 *	0.0738

C1–C6—condition 1 to condition 6, SOM—somatosensory, VIS—visual, VEST—vestibular, PREF—preference, COMP—composite. The *p*-values representing statistical significance are marked (*).

**Table 3:** Correlations between sensory organization test (SOT) results and dizziness handicap inventory (DHI) scores in patients with unilateral vestibular schwannoma before and after surgical treatment. Statistically significant correlations evaluated using Pearson’s test are marked (*).

Before Surgery				
	DHI total	DHI P	DHI E	DHI F
C5	−0.185349	−0.275523	−0.101285	−0.156375
C6	−0.059020	−0.141718	0.023968	−0.050963
VEST	−0.162163	−0.257484	−0.078064	−0.132280
COMP	−0.258424	−0.331737 *	−0.181752	−0.227361
1-Month Follow-Up				
	DHI total	DHI P	DHI E	DHI F
C5	−0.319776 *	−0.246641	−0.276210	−0.365349 *
C6	−0.344821 *	−0.337333 *	−0.323859 *	−0.323326 *
VEST	−0.309968 *	−0.236351	−0.265060	−0.358176 *
COMP	−0.401804 *	−0.370288 *	−0.366734 *	−0.401167 *
3-Month Follow-Up				
	DHI total	DHI P	DHI E	DHI F
C5	−0.326525 *	−0.203494	−0.329890 *	−0.372680 *
C6	−0.463792 *	−0.394848 *	−0.471198 *	−0.453606 *
VEST	−0.325222 *	−0.201661	−0.330222 *	−0.370529 *
COMP	−0.485413 *	−0.391527 *	−0.487078 *	−0.494936 *

DHI—dizziness handicap inventory; P—physical; E—emotional; F—functional; SOT—sensory organization test; C—condition; VEST—vestibular; COMP—composite.

**Table 4:** Correlations between video head impulse test (vHIT) gain for the lateral semicircular canal (LSC) on the affected side and dizziness handicap inventory (DHI) scores in patients with unilateral vestibular schwannoma before and after surgical treatment.

Before Surgery				
	DHI total	DHI P	DHI E	DHI F
LSC gain	−0.037547	−0.023221	0.008396	−0.078043
1-Month Follow-Up				
	DHI total	DHI P	DHI E	DHI F
LSC Gain	0.251182	0.285229	0.220319	0.219873
3-Month Follow-Up				
	DHI total	DHI P	DHI E	DHI F
LSC Gain	0.065944	−0.062073	0.153171	0.074767

DHI—dizziness handicap inventory; P—physical; E—emotional; F—functional; vHIT—video head impulse test; LSC—lateral semicircular canal.

**Table 5:** Correlations between video head impulse test (vHIT) gain for the lateral semicircular canal (LSC) on the affected side and sensory organization test (SOT) results in patients with unilateral vestibular schwannoma before and after surgical treatment. Statistically significant correlations evaluated using Pearson’s test are marked (*).

Before Surgery				
	Condition 5	Condition 6	VEST	COMP
LSC gain	0.395732 *	0.270081	0.396251 *	0.409867 *
1-Month Follow-Up				
	Condition 5	Condition 6	VEST	COMP
LSC gain	−0.244589	−0.293947	−0.237798	−0.347993 *
3-Month Follow-Up				
	Condition 5	Condition 6	VEST	COMP
LSC gain	−0.334801 *	−0.186961	−0.348148 *	−0.259510

vHIT—video head impulse test; LSC—lateral semicircular canal; SOT–sensory organization test; VEST—vestibular ratio; COMP—composite.

## Data Availability

The raw data supporting the conclusions of this article will be made available by the authors on request.

## Supplementary Materials

The following supporting information can be downloaded at: https://www.mdpi.com/article/10.3390/jcm14020585/s1.
