# Supplementary material for: Functional Outcome and Balance Compensation in Patients with Unilateral Vestibular Schwannoma After Surgical Treatment—Short- and Medium-Term Observation
Source: J Clin Med. 2025 Jan 17;14(2):585. doi: 10.3390/jcm14020585 (PMC11766155; doi:10.3390/jcm14020585)
Supplement: Supplementary file 1 [file jcm-14-00585-s001.zip › jcm-3377754-supplementary.pdf]

Journal of Clinical Medicine

MDPI

Supplementary

Table S1. Pre- and postoperative results of the analyzed patients with unilateral vestibular schwannoma before and after surgical treatment – detailed data.

Before the surgery					
**DHI**	mean	median	min	max	SD
total score (0-100 points)	24.36	20.00	0.00	84.00	24.35
P subscale (0-28 points)	8.09	6.00	0.00	24.00	7.75
E subscale (0-36 points)	6.31	2.00	0.00	28.00	7.65
F subscale (0-36 points)	9.96	6.00	0.00	34.00	10.26
**vHIT**	mean	median	min	max	SD
LSC gain tumor's side	0.97	0.99	0.18	1.53	0.29
LSC gain healthy side	1.16	1.12	0.70	2.21	0.26
**SOT**	mean	median	min	max	SD
C5 (0-100)	36.63	38.67	0.00	72.00	22.39
C6 (0-100)	32.41	35.00	0.00	73.00	24.41
SOM ratio (C2/C1)	0.97	0.98	0.89	1.01	0.03
VIS ratio (C4/C1)	0.79	0.84	0.00	0.95	0.16
VEST ratio (C5/C1)	0.39	0.43	0.00	0.76	0.24
PREF ratio [(C3+C6)/(C2+C5)]	0.95	0.95	0.62	1.53	0.19
COMP score (0-100)	62.64	64.00	32.00	81.00	11.75
**7 days after the surgery**					
**SOT**	mean	median	min	max	SD
C5 (0-100)	15.74	11.33	0.00	61.67	19.81
C6 (0-100)	20.51	15.67	0.00	74.67	21.61
SOM ratio (C2/C1)	0.96	0.97	0.83	1.05	0.05
VIS ratio (C4/C1)	0.81	0.84	0.55	0.97	0.10
VEST ratio (C5/C1)	0.16	0.11	0.00	0.65	0.21
PREF ratio [(C3+C6)/(C2+C5)]	1.02	0.99	0.56	1.63	0.21
COMP score (0-100)	55.33	53.00	39.00	78.00	9.80
**1 month after the surgery**					
**DHI**	mean	median	min	max	SD
total score (0-100 points)	31.64	24.00	0.00	92.00	27.65
P subscale (0-28 points)	9.38	8.00	0.00	26.00	8.18
E subscale (0-36 points)	7.96	4.00	0.00	30.00	9.27
F subscale (0-36 points)	14.31	14.00	0.00	36.00	11.68
**vHIT**	mean	median	min	max	SD
LSC gain tumor's side	0.52	0.46	0.01	1.44	0.27
LSC gain healthy side	1.05	1.05	0.61	1.69	0.21
**SOT**	mean	median	min	max	SD
C5 (0-100)	40.82	47.33	0.00	76.33	25.33
C6 (0-100)	38.94	38.67	0.00	82.00	24.98
SOM ratio (C2/C1)	0.96	0.97	0.87	1.02	0.03
VIS ratio (C4/C1)	0.84	0.86	0.55	1.05	0.11
VEST ratio (C5/C1)	0.43	0.51	0.00	0.80	0.27
PREF ratio [(C3+C6)/(C2+C5)]	0.98	0.96	0.62	1.69	0.19
COMP score (0-100)	66.09	67.00	39.00	87.00	11.66
**3 months after the surgery**					

DHI	mean	median	min	max	SD
total score (0-100 points)	28.62	22.00	0.00	92.00	27.22
P subscale (0-28 points)	9.38	10.00	0.00	26.00	7.83
E subscale (0-36 points)	7.24	2.00	0.00	30.00	9.39
F subscale (0-36 points)	12.00	10.00	0.00	36.00	11.26
**vHIT**	mean	median	min	max	SD
LSC gain tumor's side	0.56	0.44	0.12	1.53	0.30
LSC gain healthy side	1.06	1.00	0.63	1.59	0.22
**SOT**	mean	median	min	max	SD
C5 (0-100)	45.26	52.00	0.00	83.33	23.46
C6 (0-100)	47.21	51.00	0.00	87.67	23.62
SOM ratio (C2/C1)	0.95	0.96	0.59	1.01	0.07
VIS ratio (C4/C1)	0.85	0.91	0.22	0.97	0.14
VEST ratio (C5/C1)	0.48	0.59	0.00	0.88	0.25
PREF ratio [(C3+C6)/(C2+C5)]	1.00	0.99	0.19	1.65	0.22
COMP score (0-100)	68.58	72.00	25.00	90.00	13.63

DHI – Dizziness Handicap Inventory; P – physical; E – emotional; F – functional; vHIT – video Head Impulse Test; LSC – lateral semicircular canal; SOT– sensory organization test;
C – Condition; SOM – somatosensory; VIS – visual; VEST – vestibular; PREF – visual preference; COMP – composite; min –minimum; max – maximum; SD – standard deviation; cm – centimeters; mm – millimeters.

Table S2. Results of the Dizziness Handicap Inventory (DHI) questionnaire in 45 patients with unilateral vestibular schwannoma before and after the surgery. Pre- and postoperative handicap categories (A) and evaluation of change of the result after the surgery (B) according to the Whitney method.

A	*Before surgery (n; %)*	*1 month after surgery (n; %)*	*3 months after surgery (n; %)*
light handicap (DHI =<30)	28; 62.22%	26; 57.78%	30; 66.67%
avarage handicap (DHI= 31-60)	13; 28.89%	11; 24.44%	9; 20%
severe handicap (DHI= 61-100)	4; 8.89%	8; 17.78%	6; 13.33%
**B**	Before surgery vs 1 month after surgery (n; %)	Before surgery vs 3 months after surgery (n; %)	1 month vs 3 months after surgery (n; %)
DHI decrease min 18 points	6; 13.33%	7; 15.56%	7; 15.55%
DHI increase min 18 points	13; 28.89%	14; 31.11%	3; 6.67%
DHI without clinically important changes	26; 57.78%	24; 53.33%	35; 77.78%

DHI – Dizziness Handicap Inventory; n – number of patients; %- percentage of patients.
